# Hepatitis B Immunization Status in Children with Chronic Kidney Disease: Experience at a Single Center, Egypt

**DOI:** 10.3390/jcm12051864

**Published:** 2023-02-27

**Authors:** Doaa Mohammed Youssef, Amal S. El-Shal, Rabab M. Elbehidy, Mohamed Adel Fouda, Sally M. Shalaby, Lamiaa Lotfy El Hawy, Abdelrahman Fathi Elsadek, Mayy Abd Alfattah Neemat-Allah, Seham M. Ramadan, Amal Gohary, Faika Arab, Mona Alsharkawy, Sabry Abdel Rahman Tolba, Mohamed Mohamed Abdelsalam, Ezzat Kamel Amin, Mona Hamed Gehad

**Affiliations:** 1 Pediatrics Department, Faculty of Human Medicine, Zagazig University, Zagazig 44519, Egypt; 2 Medical Biochemistry Department, Faculty of Human Medicine, Zagazig University, Zagazig 44519, Egypt; 3 Medical Biochemistry and Molecular Biology Department, Armed Forces College of Medicine (AFCM), Cairo 11774, Egypt; 4 Public Health and Community Medicine, Faculty of Medicine, Zagazig University, Zagazig 44519, Egypt

**Keywords:** hemodialysis, hepatitis B virus, vaccine, children, seroconversion

## Abstract

***Background:*** Children with chronic kidney disease (CKD), particularly those who require hemodialysis (HD), are at high risk of hepatitis B virus (HBV) infection. The HBV vaccine non-/hypo-response rate among HD children remains high, and it is critical to investigate the influencing factors and their linkages. The aim of this study was to identify the pattern of HB vaccination response in HD children and to analyze the interference of various clinical and biomedical factors with the immunological response to HB vaccination. ***Methods:*** This cross-sectional study included 74 children on maintenance hemodialysis, aged between 3 and 18 years. These children were subjected to complete clinical examination and laboratory investigations. ***Results:*** Out of a total of 74 children with HD, 25 (33.8%) were positive for the HCV antibody. Regarding the immunological response to hepatitis B vaccine, 70% were non-/hypo-responders (≤100 IU/mL) and only 30% mounted a high-level response (more than 100 IU/mL). There was a significant relation between non-/hypo-response and sex, dialysis duration, and HCV infection. Being on dialysis for more than 5 years and being HCV Ab-positive were independent variables for non-/hypo-response to HB vaccine. ***Conclusions:*** Children with CKD on regular HD have poor seroconversion rates in response to the HBV vaccine, which were influenced by dialysis duration and HCV infection.

## 1. Introduction

Infections with the hepatitis B virus (HBV) are a serious global health issue. It is a major cause of liver cirrhosis and hepatocellular carcinoma [1]. HBV infections are a common cause of viral hepatitis and affect more than 257 million people worldwide [2]. The prevalence of HBV infection and exposure varies greatly with age, race/ethnicity, and birthplace [3]. The prevalence of HBV is estimated to be 6.7% among the general population in Egypt [4], with approximately 700,000 deaths occurring every year, mostly from the associated liver diseases [5].

Patients with chronic kidney disease (CKD), particularly those who require hemodialysis, are at high risk [6]. Shared hemodialysis equipment, greater exposure to blood products, frequent skin breaches, and an impaired immune system all contribute to an elevated risk of HBV infection in these patients [7]. As has been previously and more recently said, active vaccination is an important part of the preventative therapy of patients with CKD [8,9]. Vaccination against hepatitis B has been available since the early 1980s. The entire vaccination series provides 95% protection against HBV infection [10].

Vaccines produced by recombinant DNA technology have since been introduced. The use of a recombinant HBV vaccine containing hepatitis B surface antigen (HBsAg) is associated with a high rate of seroconversion [11]. In HBV vaccination, neutralizing antibodies against HBsAg indicate previous HBV infection or a triggered immune response against HBsAg [12]. HBV exposure is defined as the presence of HBsAg with or without antibodies for the hepatitis B e antigen (HBeAg) and the hepatitis B core antigen (HBcAg) [13]. A subset of patients may only have IgM class antibodies for HBcAg [14]. The appearance of antibodies for HBsAg (antiHBs) in the absence of HBsAg, HBeAg, HBcAg, and undetectable HBV DNA is referred to as a seroconversion of HBV [15]. People who were immune due to natural infections tested positive for anti-HBs and anti-HBc but negative for HBsAg. Vaccination-induced immunity was defined as samples that were positive for anti-HBs but negative for anti-HBc and HBsAg [16]. Table 1 summarizes the interpretations of serologic results.

After completing the vaccine course, patients with chronic kidney disease have lower rates of seroprotection and lower antibody titers; additionally, anti-HBs titers fall logarithmically over time. The reasons for the drop in these titrations are unknown, although they could be related to age; gender; being overweight; positive serologic statuses for HCV and human immunodeficiency virus infection; blood transfusion history; interleukin genotypes; possession of the major histocompatibility complex haplotypes HLA-B8, SCOI, or DR3; and inappropriate nutritional status, which have all been proposed as reasons for the poor immunogenicity of HB vaccines in advanced CKD [17].

Even though the response in patients with chronic kidney failure is poor, with up to 40% of patients being found as non-responders in some studies, routine vaccinations of patients and healthcare workers, as well as the use of erythropoietin instead of blood transfusions have significantly reduced the prevalence of HBV infection in patients on hemodialysis [18].

Post-vaccinal serological testing is not recommended for healthy people due to the vaccine’s excellent efficiency in establishing protective titers. Individuals who belong to at-risk groups, on the other hand, must have a serological evaluation of their levels of antibodies against the hepatitis B surface antigen (anti-HBs), and they will be considered protected if their anti-HBs levels are ≥10 IU/mL [19].

No data are available regarding the hepatitis B immunization status in patients with CKD in Egypt. The purpose of this study was to determine the status of hepatitis B vaccination among patients with CKD and to determine the risk factors for non-responsiveness to hepatitis B vaccination.

## 2. Patient and Method

### 2.1. Study Group

This cross-sectional, observational study was conducted in the Pediatrics Nephrology ward of Zagazig University Children’s Hospital during the period from October 2021 to March 2022. Seventy-four children with ESRD on maintenance hemodialysis, aged between 3–18 years, were enrolled, with a definite history of receiving the primary immunization for HBV at infancy. Egypt began a mandatory childhood vaccination program in 1992, in accordance with WHO recommendations. The Egyptian Ministry of Health prescribed three doses of yeast recombinant hepatitis B vaccine (10 µg) to all infants at the ages of two, four, and six months [20].

Patients positive for the hepatitis B surface antigen (HBsAg); those receiving immunosuppressive drugs or other immunocompromised conditions not related to ESRD; and finally, those receiving any additional HBV booster vaccination since primary vaccination were excluded. The levels of antibodies for the hepatitis C virus (HCV) were also measured.

This study was approved by the ethical committee of the Faculty of Medicine at Zagazig University within the framework of the project by the Science and Technological Development Fund (STDF) No. 28963, and written consent was taken from every subject in the study or their caregiver.

All study participants or their caregivers were subjected to a thorough history check, and patient characteristics including HBV vaccination status, age, gender, socioeconomic status, duration of dialysis, and underlying etiology of CKD were collected; anthropometric measurements including dry weight and length were used for calculations of the body mass index (BMI) in Kg/m^2^, with a full clinical examination performed.

### 2.2. Sample Collection

The blood samples were taken by venipuncture under complete aseptic conditions before any drugs were administered and were divided in two tubes (EDTA and plain for serum). Concerning the sera, the blood was allowed to coagulate at room temperature for 10–20 min; it was centrifuged for 20 min at a speed of 2000–3000 rpm; and then, the supernatant was removed. Hemolytic or contaminated sera were not used.

The specimens were stored frozen at −20 °C until testing. Routine laboratory tests including complete blood count (CBC), liver function tests (serum alanine transaminase (ALT), aspartate transaminase (AST), and serum albumin were measured by colorimetric assays (Spin react, Santa Coloma, Spain). Furthermore, renal function tests, including blood urea and serum creatinine, were estimated using colorimetric assays (Spin react, Santa Coloma, Spain). Parathyroid hormone (PTH) values were determined using a quantitative enzyme-linked immunosorbent assay (ELISA) kit provided by (Abcam com., Waltham, MA, USA), and ferritin concentrations were estimated. The detection of HCV-Ab was performed using an ELISA, markers for HBV infection included the detection of HBsAg and the measurement of antibodies for the HBsAb (anti-HBs) titer using a sandwich enzyme-linked immunosorbent assay (ELISA) (Roche Diagnostics, Basel, Switzerland; My BioSource, San Diego, CA, USA, respectively) according to the manufactures’ instruction to detect infected cases and the immune protection level of the subjects studied.

We estimated the serum HBsAb concentration using the sandwich ELISA method according to the manufacturer’s instructions (AccuDiag™- HBsAb Quantitative ELISA kit, CA, USA). A total of 50 µL of all standard samples were added into their respective wells except the blank sample. After that, 50 µL of the streptavidin–horseradish peroxidase (HRP) conjugate was added into each well except the blank sample and mixed by tapping the plate gently. Then, the plate was incubated while covered for 60 min at 37 °C. At the end of the incubation, each well was washed 5 times with a diluted wash buffer. Each time, the microwells were allowed to soak for 30–60 s. After the final washing cycle, 50 µL of the chromogen A solution and 50 µL of the chromogen B solution were added into each well, including the blank sample, and the plate was incubated at 37 °C for 15 min, avoiding light. The enzymatic reaction between the chromogen solutions and the HRP conjugate becomes blue in the calibration curve standard wells (except for 0 mIU/mL) and in the anti-HBs-positive sample wells. Finally, 50 µL of the stop solution was added into each well and mixed gently. The blue solution turns yellow after the reaction stops. The absorbance of the resulting product was measured spectrophotometrically at 450 nm. The absorbance was proportional to the concentration of HBsAb. A standard curve was constructed by plotting the absorbance value versus the HBsAb concentration of the standards, and the concentrations of the unknown samples were determined using this standard curve. Non-/hypo-responses and high-level responses were defined as anti-HBs ≤ 100 IU/mL and >100 IU/mL, respectively.

### 2.3. Statistical Analysis

The data were analyzed using the Statistical Package of Social Science (SPSS) program for Windows (Standard version 20, Chicago, IL, USA). The normality of the data was first tested with a one-sample Kolmogorov–Smirnov test. The qualitative data were described as a number and a percent. The continuous variables were presented as mean ± SD (standard deviation). The association between categorical variables was tested using a Chi-square test. The two groups were compared with the Mann–Whitney U test for non-parametric data. A logistic regression was used to identify independent predictors of immunological response to the hepatitis B vaccine among the samples studied. The tests were significant when *p* ≤ 0.05.

## 3. Results

### 3.1. Demographic and Clinical Characteristics of Studied Sample

The total number of patients on hemodialysis was 74. Their ages ranged from 3 to 18, with an average age of 13.46 + 3.71 years. Their weights ranged from 12 to 58 kg, with a mean weight of 32.93 ± 11.792 kg. The body mass index ranged from 11 to 45 with a mean was 19.28 ± 5.90. Most of them were female, 59.5%, and on dialysis for more than 5 years, 56.8%, and 25 (33.8%) were positive for the HCV antibody. The main underlying causes were chronic glomerulonephritis, unknown, and obstructive uropathy: 33.8%, 31.1%, and 29.7%, respectively. Regarding immunological response to hepatitis B vaccine, 70% were non-/hypo-responders (≤100 IU/mL) and only 30% mounted a high-level response (more than 100 IU/mL) (Table 2).

### 3.2. The Relationship between Some Demographic and Clinical Factors and the Immune Response to the Hepatitis B Vaccine in the Study Group

There was a statistically significant relation between non-/hypo-response and sex, dialysis duration, and HCV infection (*p* = 0.0108, *p* = 0.021, and *p* = 0.0035 *), respectively, where being male, being on dialysis for more than 5 years, and have an HCV Ab-positive mounted non-/hypo-response corresponded to 86.7%, 81.0%, and 92.0%, respectively. There was no statistically significant difference between high-level responders and non-/hypo-responders with respect to age, BMI, serum albumin level, ferritin, creatinine, and PTH level (Table 3).

### 3.3. The Immunological Response to HBV in Relation to Causes of Chronic Renal Diseases

**Figure 1:**
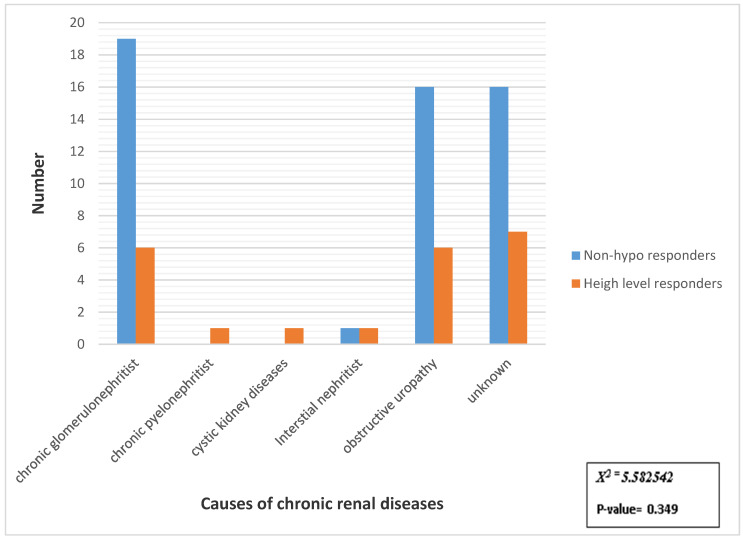
Distribution of samples studied according to immunological response to HBV in relation to causes of chronic renal diseases. X^2^ = Chi-square test.

There was a statistically insignificant relation between the cause of chronic renal diseases and immunological response to HBV (Figure 1).

### 3.4. Multivariate Analysis of Some Demographic and Clinical Data Predictors for Non-/Hypo-Response to Hepatitis B Vaccine

Being on dialysis for more than 5 years and being HCV Ab-positive were independent variables for HB vaccination non-/hypo-response (AOR = 17.610, *p* = 0.002 and AOR = 8.945, *p* = 0.002 *), respectively. However, being of the male sex was a non-significant independent factor for non-/hypo-response to the HB vaccine (AOR = 3.499, *p* = 0.074) (Table 4).

## 4. Discussion

Patients undergoing chronic hemodialysis (HD) are particularly prone to HBV infection. After infection, they are more prone than the general population to become chronic carriers. As a result, they could become an infection source for other patients and medical professionals [21].

Patients who test positive for HBsAg should receive hemodialysis in a separate room reserved exclusively for patients positive for HBsAg. They should use specialized machines, equipment, and materials, and staff members should not care for both patients who are HBsAg positive and susceptible at the same time (shift) or in the treatment area. Dialyzers should not be used again on patients positive for HBsAg because HBV is easily transmitted through occupational blood exposure, and reusing dialyzers from patients positive for HBsAg may expose staff who are susceptible to HBV to infection. Patients chronically infected with HBV are contagious and at risk of chronic liver disease [22].

In studies that used techniques to boost vaccine effectiveness, the immunological effect of the hepatitis B vaccine was improved to some extent. However, the proportion of non-/hypo-response remains significant, and the vaccine is ineffective in protecting patients against HBV infection [23]. It is critical to figure out the HB vaccination response pattern in patients on hemodialysis and to investigate the elements that influence non-response/hypo-response to the hepatitis B vaccine in patients on HD. Therefore, to give more precise prevention and control methods of non-/hypo-response to hepatitis B vaccine, we explored the factors impacting non-/hypo-response to hepatitis B vaccine.

Regarding immunological response to hepatitis B vaccine, 70% were non-/hypo-responders (≤100 IU/mL) and only 30% mounted high-level response (more than 100 IU/mL); this agrees with the results of Almueilo and Samir H [24], who found that seroconversion to the HB vaccination was attained in 70.3 % out of 101 patients on HD and that only 49 patients (48.5%) had a strong reaction with a HBsAb level of >100 IU/mL, while Al Saran et al. [25], in their study, aimed to determine the response to HBV vaccination in patients on HD and to identify the factors that could affect this response, reporting that 129 out of 144 (89.64%) patients were responders (anti-HBs ≥ 10), whereas non-responders (anti-HBs < 10 IU/l) made up 15 out of 144 (10.4%) patients. The seroprotection rates in patients on chronic HD following HB immunization have varied. A range of factors, including sample size, age, weight, and gender distribution, might contribute to such inequalities: the prevalence of HCV infection; duration on HD; effectiveness of HD therapy; and presumably, genetic differences amongst populations. Varying the dose and scheduling regimens for the vaccine contribute to the HD population’s variable response rate.

There was no statistically significant difference between high-level responders and non-/hypo-responders with respect to age. In agreement with our result, other studies revealed that age did not affect immune response to the HB vaccine [10,26]. This is in contrast with other studies that found that a positive vaccine response was predicted by being younger [24,27,28], which could be attributed to age-related immune status changes. This effect was not observed in our study because our patients on dialysis were younger than those in the other studies.

Males were less likely than females to mount a protective response after vaccination, indicating that gender was a contributing factor. This result was consistent with that in the literature [23,25,29]. In some circumstances, antibody induction in males was thought to be sex related. Females typically have stronger innate, humeral, and cellular immune responses to viral infections and vaccination than males. In general, estrogens stimulate the immune system, whereas androgens suppress it. Sex hormones, such as androgen, can, however, directly interact with the HBV genome that has been integrated into the cell nucleus and can activate transcription of HBV oncoproteins [30]. This contrasts with another study that found no statistically significant difference between responders and non-responders when it came to gender [24].

The duration of dialysis has been linked to a deleterious impact on immunological responses to the hepatitis B vaccine. In agreement with our study, Ayub et al. [18] followed-up with 83 patients on HD and collected quantitative serologic measurements every 2 months over a 1-year period to determine HB vaccine immune response in these patients. All patients displayed decreasing antibody titers during the observation period. Moreover, anti-HBs titers in good responders dropped to unprotected levels after one and two years in these patients. Long-term dialysis may have an impact on the immune system, which could alter the responses of patients on HD to the hepatitis B vaccine. Furthermore, due to the frequent requirement for blood product transfer and the likelihood of utilizing contaminated dialysate or dialysis materials, patients on dialysis are at an increased risk of viral transmission [31]. Furthermore, linear macromolecular straight-chain molecules can be partially transmitted through the dialysis membrane, according to studies. Natural antibodies have a non-linear shape, and the antibodies produced by the recombinant hepatitis B vaccine may differ from natural antibodies, allowing them to flow through the dialysis membrane more easily, resulting in more antibodies being lost [32]. In contrast to our findings, some investigations have found that vaccination status is unrelated to the duration of CKD [10,25].

In the current study, the response to the vaccine in 25 patients with HCV infections was significantly different from patients negative for HCV. Similar to the findings of the current study, some studies observed a lower response rate in patients with HCV who are on HD [33,34]. However, some studies reported that HCV infection did not influence the response to HB vaccination in their group of patients on HD [24].

Co-infection of HCV/HBV is common in endemic areas and among people at risk of parenterally transmissible infections [35]. The reason for the reduced vaccine response in people infected with HCV appears to be multifactorial. Patients with HCV who are HBV vaccine non-responders were found to have increased PD-1 expression, a negative immune modulator associated with decreased T cell activation in response to both general and virus-specific stimulation [36]. An HCV core was found to strongly inhibit the cytotoxic immune response in a murine model [37,38]. Finally, impairment in the humoral arm of the immune system has been observed in patients with HCV, which has been linked to poor HBV vaccine response [39]. Overall, the presence of these immune changes in individuals infected with HCV is likely to play a significant role in vaccine hypo-responsiveness, and the extent to which immune response is reduced could be a factor related to the disparity between some of the studies. Genetic variation may also be linked to the vaccine response discrepancy, with genetically determined low responsiveness to HBsAg being reported in carriers of various HLA types such as B8, B44, DR3, DR7, and DQ2, but this is less likely to be a major contributor to the response discrepancy among patients with HCV [40,41].

There was no statistically significant difference between a high-level response and non-/hypo-response with respect to serum albumin level. These results were in concordance with studies reporting that there was no noticeable difference between those who responded to the HB vaccine and those who did not, with regard to serum albumin levels [24,25]. However, other study revealed a significant positive correlation between anti-HBs titer and serum albumin levels in the HD subgroup [27,42].

In the present study, the primary cause of ESRD did not affect the response to the hepatitis B vaccine. These results agree with those reported by Al Saran et al. [25].

There was also no statistically significant difference between high-level response and non-/hypo-response with respect to serum creatinine level. This result agreed with those of Hashemi et al. [43], who detected no significant correlation between anti-HBs titers and serum creatinine. In contrast, Mohammed et al. revealed a highly significant negative correlation between anti-HBs titers and serum creatinine [27].

A majority of people exhibiting non-/hypo-response to the hepatitis B vaccine were those on dialysis for more than 5 years and those who were HCV Ab-positive. Our findings are critical in providing scientific data for the precise prevention and control of non-/hypo-response to the hepatitis B vaccine in patients on HD. However, being of the male sex was a non-significant independent factor for non-/hypo-response to the HB vaccine. Another study found that being male and being on dialysis longer were independent risk factors for non-/hypo-response to hepatitis B immunization [23].

Hepatitis B management and prevention depend largely on increasing testing, awareness, and access to healthcare services, as well as on preventative measures such as hepatitis B vaccination in high-risk populations. Lowering the burden of hepatitis B is dependent on vaccinating high-risk populations, receiving a diagnosis, being aware of their status, and being able to access medical care and treatment as needed. Action plans enacted by important public health authorities and partners can be guided by ongoing monitoring of HBV prevalence, immunity, and susceptibility [44].

## 5. Conclusions

Children with CKD on regular HD have poor seroconversion rates in response to the HBV vaccine, which were influenced by dialysis duration and HCV infection. The patients’ response to the immunization was unaffected by a variety of demographic and chemical parameters, including albumin and PTH levels.

## 6. Recommendations

A working strategy to improve the rate of successful immunization against HB in patients on chronic HD would be to identify individuals with chronic kidney disease (CKD) at an earlier stage, such as Stages 2 and 3, and to offer immunization to patients who are neither infected with nor immune to the virus. We recommend that future studies include more centers and that more factors influencing the vaccination status be studied. Patients with CKD should have annual screenings to check for antiHBs titer decreases and to receive additional HBV vaccination doses.

## 7. Study Limitations

First, it was proposed that patients on HD should be vaccinated prior to starting dialysis. However, because our study only looked at patients on HD, the next phase in the research should be to study people who are not yet on dialysis. Second, it is possible that our sample size is insufficient. As a result, more studies based on a larger survey sample may be required to establish the duration of seroprotection.

## Figures and Tables

**Table 1:** Interpretations of serologic markers of hepatitis B virus.

HBsAg	Total anti HBc	IgM anti HBc	Anti HBs	Interpretation
−	−	−	−	Not infected
+	−	−	−	Acute infection (early phase)
+	+	+	−	Acute infection
−	+	+	−	Recovering from acute infection
−	+	−	+	İmmunized patient with a past infection
+	+	−	−	Chronic infection
−	+	−	−	Chronic infection with low-level viremia or false positive
−	−	−	+	Immunized

HBsAg: hepatitis B virus surface antigen; antiHBs: antibodies for HBsAg; antiHBc: antibody against hepatitis B core antigen; +: seropositive; −: seronegative.

**Table 2:** Demographic and clinical characteristics of the samples studied.

Variables	Study Group (*n* = 74)	
	Mean ± SD	Min–Max
-Age (years)	13.46 + 3.71	3–18
-Weight (kg)	32.93 + 11.792	12–58
-Body mass index (BMI)	19.28 + 5.90	11–45
-PTH (pmol/L)	254.0 + 237.69	22–937
-Serum albumin (g/dL)	4.24 + 0.569	2–5
-Serum ferritin (μg/L)	521.72 + 320.12	34–987
-Serum creatinine (mg/dL)	7.06 ± 2.124	1–11
	**No (%)**	**OR (95% C.I. Lower–Upper)**
**Sex:** -Male -Female	30 (40.5%) 44 (59.5%)	0.4 (0.29–0.53) 0.6 (0.47–0.71)
**Dialysis duration:** -Less than or equal 5 y -More than 5 y	32 (43.2%) 42 (56.8%)	0.4 (0.32–0.56) 0.5 (0.45–0.68)
**Hepatitis C virus antibody:** -Positive -Negative	25 (33.8%) 49 (66.2%)	0.3 (0.24–0.47) 0.6 (0.53–0.76)
**Primary renal disease:**		
-Chronic glomerulonephritis	25 (33.8%)	0.3 (0.22–0.45)
-Chronic pyelonephritis	1 (1.4%)	0.01 (0.00–0.07)
-Cystic kidney diseases	1 (1.4%)	0.01 (0.00–0.07)
-Interstitial nephritis	2 (2.7%)	0.03 (0.003–0.095)
-Obstructive uropathy	22 (29.7%)	0.30 (0.19–0.42)
-Unknown	23 (31.1%)	0.31 (0.21–0.43)
**Immunological responses to hepatitis B vaccine:** -Non-/hypo-response -High-level response	52 (70%) 22 (30%)	0.7 (0.59–0.80) 0.3 (0.19–0.42)

PTH: parathyroid hormone; BMI: body mass index.

**Table 3:** Relation between some demographic and clinical characteristics and immunological response to hepatitis B vaccine among the sample studied.

Variables	Immunological Response		*p*-Value
	The Non-/Hypo-Response (*n* = 52)	High-Level Response (*n* = 22)	
-Age (years) (Median) (Min–Max)	14 (3–18)	16 (6–18)	0.11 ^#^
-Weight (kg) (Median) (Min–Max)	34 (12–58)	35 (16–50)	0.74 ^#^
-BMI (Median) (Min–Max)	19 (11–37)	18 (11–45)	0.91 ^#^
-PTH (pmol/L) (Median) (Min–Max)	197.5 (22–1456)	181.5 (31–1466)	0.33 ^#^
-Serum albumin (g/dL) (Median) (Min–Max)	4 (2–5)	4 (3–5)	0.48 ^#^
-Serum ferritin (μg/L) (Median) (Min–Max)	763 (34–1800)	670 (48–1811)	0.13 ^#^
-Serum creatinine (mg/dL) (Median) (Min–Max)	7.25 (2–11)	7 (1–9)	0.72 ^#^
	**N (%)**	**N (%)**	***p*-value**
**Sex:** - Male (30) - Female (44)	26 (86.7%) 26 (59.1%)	4 (13.3%) 18 (40.9%	X^2^0.01 *
**Dialysis duration:** - Less than or equal 5 y (32) - More than 5 y (42)	18 (56.2%) 34 (81.0%)	14 (43.8%) 8 (19.9%)	X^2^0.02 *
**HCV (Ab):** - Positive (25) - Negative (49)	23 (92.0%) 29 (59.2%)	2 (8.0%) 20 (40.8%)	X^2^0.004 *

# Mw = Mann–Whitney U test, X^2^ = Chi-square test, * = statistically significant., BMI: body mass index, PTH: parathyroid hormone.

**Table 4:** Multivariate analysis of some demographic and clinical data predictors for no or inadequate response to hepatitis B vaccine.

Variables	Univariate Analysis		Multivariate Analysis	
	Unadjusted Odds Ratio (95% C.I. Lower–Upper)	*p*-Value	Adjusted Odds Ratio (95% C.I. Lower–Upper)	*p*-Value
**-Sex (male)**	4.5 (1.34–15.23)	0.010 *	3.5 (0.88–13.85)	0.07
**-Dialysis duration (more than 5 years)**	0.3 (0.11–0.86)	0.021 *	17.6 (2.85–108.96)	0.002 *
**-HCV (Ab) positive**	7.9 (1.68–37.49)	0.004 *	8.9 (2.24–35.75)	0.002 *

* = statistically significant.

## Data Availability

The data that support the findings of this study are available from the corresponding author upon reasonable request.

## References

[1] Ali M., Idrees M., Ali L., Hussain A., Rehman I.U., Saleem S., Afzal S., Butt S. (2011). Hepatitis B virus in Pakistan: A systematic review of prevalence, risk factors, awareness status and genotypes. Virol. J..

[2] Samsunder N., Ngcapu S., Lewis L., Baxter C., Cawood C., Khanyile D., Kharsany A.B. (2019). Seroprevalence of hepatitis B virus: Findings from a population-based household survey in KwaZulu-Natal, South Africa. Int. J. Infect. Dis..

[3] Le M.H., Yeo Y.H., Cheung R., Henry L., Lok A.S., Nguyen M.H. (2020). Chronic hepatitis B prevalence among foreign-born and US-born adults in the United States, 1999–2016. Hepatology.

[4] Lehman E.M., Wilson M.L. (2009). Epidemiology of hepatitis viruses among hepatocellular carcinoma cases and healthy people in Egypt: A systematic review and meta-analysis. Int. J. Cancer.

[5] World Health Organization (2021). Global Progress Report on HIV, Viral Hepatitis and Sexually Transmitted Infections, 2021: Accountability for the Global Health Sector Strategies 2016–2021: Actions for Impact: Web Annex 2: Data Methods.

[6] Grzegorzewska A.E. (2012). Hepatitis B vaccination in chronic kidney disease: Review of evidence in non-dialyzed patients. Hepat. Mon..

[7] Somi M.H., Hajipour B. (2012). Improving hepatitis B vaccine efficacy in end-stage renal diseases patients and role of adjuvants. Int. Sch. Res. Not..

[8] Krueger K.M., Ison M.G., Ghossein C. (2020). Practical guide to vaccination in all stages of CKD, including patients treated by dialysis or kidney transplantation. Am. J. Kidney Dis..

[9] Garthwaite E., Reddy V., Douthwaite S., Lines S., Tyerman K., Eccles J. (2019). Clinical practice guideline management of blood borne viruses within the haemodialysis unit. BMC Nephrol..

[10] Amjad A., Kumar J., Chaudary N., Kumar K., Nazar C.M.J., Khan K. (2019). Hepatitis B Vaccination status in chronic kidney disease: Experience at Pakistan institute of medical sciences. Cureus.

[11] Zhao H., Zhou X., Zhou Y.-H. (2020). Hepatitis B vaccine development and implementation. Hum. Vaccines Immunother..

[12] Simmonds P., Midgley S. (2005). Recombination in the genesis and evolution of hepatitis B virus genotypes. J. Virol..

[13] Chu C.-J., Keeffe E.B., Han S., Perrillo R.P., Min A.D., Soldevila-Pico C., Carey W., Brown R.S., Luketic V.A., Terrault N. (2003). Prevalence of HBV precore/core promoter variants in the United States. Hepatology.

[14] Gaeta G.B., Stornaiuolo G., Precone D.F., Lobello S., Chiaramonte M., Stroffolini T., Colucci G., Rizzetto M. (2003). Epidemiological and clinical burden of chronic hepatitis B virus/hepatitis C virus infection. A multicenter Italian study. J. Hepatol..

[15] Lok A., McMahon B.J. (2007). Chronic hepatitis B. Hepatol. Baltim. Orlando.

[16] López-Gatell H., García-García L., Echániz-Avilés G., Cruz-Hervert L.P., Olamendi-Portugal M., Castañeda-Desales D., Sánchez-Aleman M.A., Romero-Martínez M., DeAntonio R., Cervantes-Apolinar M.Y. (2019). Hepatitis B seroprevalence in 10–25-year-olds in Mexico-the 2012 national health and nutrition survey (ENSANUT) results. Hum. Vaccines Immunother..

[17] Fabrizi F., Cerutti R., Dixit V., Ridruejo E. (2021). Hepatitis B virus vaccine and chronic kidney disease. The advances. Nefrologia.

[18] Ayub¹ M.A., Bacci M.R., Fonseca F.L.A., Chehter E.Z. (2014). Hemodialysis and hepatitis B vaccination: A challenge to physicians. Int. J. Gen. Med..

[19] Costa N.C.P.d., Canhestro M.R., Soares C.M.B.M., Rodrigues J.S. (2017). Monitoring of post-vaccination anti-HBs titles vaccine in children and adolescents in the pre-dialysis of chronic kidney disease. Braz. J. Nephrol..

[20] Eladawy M., Gamal A., Fouad A., El-Faramawy A. (2015). Hepatitis B Virus Vaccine immune response in Egyptian children 15–17 years after primary immunization; should we provide a booster dose?. Egypt. J. Pediatric Allergy Immunol..

[21] Fabrizi F., Messa P., Martin P. (2008). Hepatitis B virus infection and the dialysis patient. Seminars in Dialysis.

[22] Himmelfarb J., Sayegh M.H. (2010). Chronic Kidney Disease, Dialysis, and Transplantation E-Book: A Companion to Brenner and Rector’s The Kidney.

[23] Feng Y., Wang J., Shao Z., Chen Z., Yao T., Dong S., Wu Y., Shi X., Shi J., Liu G. (2021). Predicting related factors of immunological response to hepatitis B vaccine in hemodialysis patients based on integration of decision tree classification and logistic regression. Hum. Vaccines Immunother..

[24] Almueilo S.H. (2017). Evaluation of response to hepatitis B vaccination in chronic hemodialysis patients. Saudi J. Med. Med. Sci..

[25] Al Saran K., Sabry A., Elhalawany Z., Ismail M. (2014). Factors affecting response to hepatitis B vaccine among hemodialysis patients in a large Saudi Hemodialysis Center. Saudi J. Kidney Dis. Transplant..

[26] Khashaba A., Manal S., Mohammed M., Ghada S., Rashad M. (2006). Study of immune response to hepatitis B vaccine in Egyptian preschool children. J. Viral Hepat..

[27] Mohammed H.I., El-Hefenawy S.M., Mohamed S.F. (2017). Assessment of immune response to hepatitis B virus vaccine in chronic hemodialysis patients. Menoufia Med. J..

[28] Gomes L.C., Sanson M.C.G., Brainin P., Melo M.d.C.V.d., de Souza R.M., Mazaro J., Lima K.O., Resende J.S., Vieira I.V.M., Mesquita E.d.S. (2021). Levels of hepatitis B antibody titers are affected by age and doses gap time in children from a high endemic area of the western Amazon. PLoS ONE.

[29] Sari F., Taskapan H. (2012). Good response to HBsAg vaccine in dialysis patients is associated with high CD4+/CD8+ ratio. Int. Urol. Nephrol..

[30] Ruggieri A., Gagliardi M.C., Anticoli S. (2018). Sex-dependent outcome of hepatitis B and C viruses infections: Synergy of sex hormones and immune responses?. Front. Immunol..

[31] Sit D., Esen B., Atay A.E., Kayabaşı H. (2015). Is hemodialysis a reason for unresponsiveness to hepatitis B vaccine? Hepatitis B virus and dialysis therapy. World J. Hepatol..

[32] Feng Y., Shi X., Shi J., Gao L., Liu G., Cheng Y., Pan M., Li C., Wang J., Guo X. (2017). Immunogenicity, antibody persistence, and safety of the 60 μg hepatitis B vaccine in hemodialysis patients: A multicenter, randomized, double-blind, parallel-controlled trial. Expert Rev. Vaccines.

[33] Elzouki A.-N., Elgamay S.M., Zorgani A., Elahmer O. (2014). Hepatitis B and C status among health care workers in the five main hospitals in eastern Libya. J. Infect. Public Health.

[34] Ashhab A.A., Rodin H., Campos M., Abu-Sulb A., Hall J.A., Powell J., Debes J.D. (2020). Response to hepatitis B virus vaccination in individuals with chronic hepatitis C virus infection. PLoS ONE.

[35] Shih Y.-F., Liu C.-J. (2020). Hepatitis C virus and hepatitis B virus co-infection. Viruses.

[36] Moorman J.P., Zhang C.L., Ni L., Ma C.J., Zhang Y., Wu X.Y., Thayer P., Islam T.M., Borthwick T., Yao Z.Q. (2011). Impaired hepatitis B vaccine responses during chronic hepatitis C infection: Involvement of the PD-1 pathway in regulating CD4+ T cell responses. Vaccine.

[37] Large M.K., Kittlesen D.J., Hahn Y.S. (1999). Suppression of host immune response by the core protein of hepatitis C virus: Possible implications for hepatitis C virus persistence. J. Immunol..

[38] Bauer T., Jilg W. (2006). Hepatitis B surface antigen-specific T and B cell memory in individuals who had lost protective antibodies after hepatitis B vaccination. Vaccine.

[39] Doi H., Iyer T.K., Carpenter E., Li H., Chang K.-M., Vonderheide R.H., Kaplan D.E. (2012). Dysfunctional B-cell activation in cirrhosis resulting from hepatitis C infection associated with disappearance of CD27-Positive B-cell population. Hepatology.

[40] Kruskall M., Alper C.A., Awdeh Z., Yunis E.J., Marcus-Bagley D. (1992). The immune response to hepatitis B vaccine in humans: Inheritance patterns in families. J. Exp. Med..

[41] McDermott A., Zuckerman J.N., Sabin C.A., Marsh S.G.E., Madrigal J.A. (1997). Contribution of human leukocyte antigens to the antibody response to hepatitis B vaccination. Tissue Antigens.

[42] Bel’eed K., Wright M., Eadington D., Farr M., Sellars L. (2002). Vaccination against hepatitis B infection in patients with end stage renal disease. Postgrad. Med. J..

[43] Hashemi B., Mahdavi-Mazdeh M., Abbasi M., Hosseini-Moghaddam S.M., Zinat N.H., Ahmadi F. (2011). Efficacy of HBV vaccination in various stages of chronic kidney disease: Is earlier better?. Hepat. Mon..

[44] Roberts H., Ly K.N., Yin S., Hughes E., Teshale E., Jiles R. (2021). Prevalence of HBV Infection, Vaccine-Induced Immunity, and Susceptibility Among At-Risk Populations: US Households, 2013–2018. Hepatology.

